# Sex, stress, and epigenetics: regulation of behavior in animal models of mood disorders

**DOI:** 10.1186/2042-6410-4-1

**Published:** 2013-01-21

**Authors:** Georgia E Hodes

**Affiliations:** 1 Fishberg Department of Neuroscience and Freidman Brain Institute, Icahn School of Medicine at Mount Sinai, New York, NY, 10029, USA

**Keywords:** Epigenetics, Stress, Depression, Anxiety, Animal models, DNA methylation, Histone modifications, Sex differences.

## Abstract

Women have a higher incidence of stress related disorders including depression and generalized anxiety disorder, and epigenetic mechanisms likely contribute to this sex difference. Evidence from preclinical research suggests that epigenetic mechanisms are responsible for both sexual dimorphism of brain regions and sensitivity of the stress response. Epigenetic modifications such as DNA methylation and histone modifications can occur transgenerationally, developmentally, or in response to environmental stimuli such as stress exposure. This review will provide an overview of the various forms of epigenetic modifications observed in the central nervous system and will explain how these mechanisms contribute to a sexually dimorphic brain. It will also discuss the ways in which epigenetic alterations coincide with, and functionally contribute to, the behavioral response to stress across the lifespan. Ultimately, this review will focus on novel research utilizing animal models to investigate sex differences in epigenetic mechanisms that influence susceptibility to stress. Exploration of this relationship reveals epigenetic mechanisms with the potential to explain sexual dimorphism in the occurrence of stress related disorders.

## Review

### Introduction

Stress-related disorders such as major depression and generalized anxiety affect over 20% of the American population within their lifetime, and have an annual prevalence approaching 10% [1]. Currently available treatments for these disorders produce remission in only 40-60% of patients [2]. Therefore, a clear need exists for novel therapeutic strategies that target the underlying biology involved with vulnerability to depression and anxiety [2]. Within the clinical populations presenting for stress-related disorders, there is a higher incidence in women compared to men [3]. One factor that may contribute to the low response to treatment has been the continued reliance on using male animals in preclinical research. A recent meta-analysis of publications indicated that 65% of studies in pharmacology and 55% of neuroscience papers used male subjects exclusively [4]. Even within the uncommon cases where females were included, only 20% of neuroscience and pharmacology manuscripts examined sex as a factor in the analysis of their data.

While females have a higher risk of developing stress-related disorders, males are at greater risk of developing early life social/cognitive behavior-related disorders such as autism spectrum disorders [5] and attention deficit disorders [6]. The consistency of the relationship between sex and age at which symptoms first occur suggest some biological predetermination of risk, although environmental experience also clearly plays a role in modulating severity, at least for stress related disorders [7]. To date, no genetic studies have demonstrated a clear biological basis for any of these disorders. In light of our inability to attribute these disorders to single polymorphisms within candidate genes, it is likely that these complex disorders are due to myriad small changes in a number of gene networks. Towards this end, a growing body of research is investigating how epigenetic mechanisms may contribute to an individual’s stress response and sex differences in brain development.

### What are epigenetic mechanisms?

Epigenetics refers to changes that alter gene transcription in the absence of direct alterations to the genome itself [8]. The term was originally used by Waddington in 1942 to describe how a phenotype was produced by an interaction of genes and their products, as it was observed that genetic variation did not necessarily match phenotypic variation [9]. Over time this definition has shifted, and epigenetics is now more commonly used to describe potentially heriable and functionally relevant modifications of gene expression and chromatin structure in the absence of changes in genetic composition [8,10].

There are a number of epigenetic mechanisms that can modulate sex differences in response to stress. These include DNA methyltransferases (Dnmts), which act directly to suppress transcription or act in concert with methyl-CpG binding domain (MBD) proteins that in turn can recruit histones to modify access of transcription factors to DNA. In addition micro RNAs are another epigenetic mechanism that contributes to sex differences in the stress response and have been reviewed previously in this journal [11]. A class of enzymes, Dnmts act by catalyzing the addition of a methyl group to cytosine 5 resulting in the formation of 5-methylcytosine (5mC) [12]. In mammals, DNA methylation generally occurs at CpG islands distributed throughout gene promoter regions. In normal mammalian development, 60-90% of CpG sites are already methylated [12]. Addition of this methyl group can lead to direct transcriptional silencing by occlusion of DNA transcription factor binding sites. DNA methylation is necessary for normal development and is involved in such varied processes as x-inactivation, imprinting, transcriptional repression, silencing of repetitive elements in the genome and cancer development [13]. The Dnmt-regulated epigenetic landscape is further complicated due to the multiple classes of Dnmts present in the brain. Dnmt 1-mediated methylation maintains methyl group placement between progenitor and daughter cells [12]. Dnmt 3a and 3b are involved in *de novo* methylation, and therefore can place methyl marks on previously non-methylated DNA [12]. Dnmt 2 and 3-like (Dnmt 3 l) belong to the Dnmt family based on sequence homology [14], but their roles in DNA methylation are poorly understood and are involved in the repression of transcription of retrotransposon elements [14] and methylating RNA[15,16] respectively.

The majority of what is known about the functional role of Dnmts comes from the literature on development. Dnmt 1, 3a and 3b are necessary for normal prenatal development as lethality occurs in the mammalian embryo following constitutive removal of Dnmt 1, Dnmt 3b or combined 3a/3b knockouts [17-19]. Dnmt 3a embryonic knockouts can survive past birth, but die within 4 weeks [19]. Conditional knockouts using floxed mice have allowed for examination of the role of Dnmts during the postnatal period [20]. Specifically, during both prenatal and early postnatal development, regulation of DNA methylation is highly implicated in the formation of the sexually differentiated brain [8]. Extending these developmental time points, recent studies have begun to examine an alternative role for these enzymes in adult animals. For example, Dnmt expression contributes to stress-related learning and memory [21] and the response to stress itself [22] through transcriptional repression. In general, changes brought about by Dnmts are thought to be stable and long lasting. However, there is recent evidence that Tet proteins act as demethylases by converting 5mC to 5-Hydroxymethylcytosine (5hmC), allowing the possibility for reversal of DNA methylation [23,24] and removal of transcriptional repression that may have functional significance.

In addition to acting directly on transcriptional targets, Dnmts work in concert with MBD proteins to alter chromatin structure through recruitment of histone modifiers [13]. The MBD protein family includes MBD1, MBD2, MBD3 and MBD4 in addition to MeCP2, all based on homology of the MBD amino acid sequence originally identified in MeCP2 [13,25]. MeCP2 is an X-linked protein [26] whose mutation is implicated in the development of Rett syndrome in humans [12]. MBD proteins, with the exception of MBD3, bind preferentially to methylated DNA [13]. The MBD proteins have numerous functional roles, including both transcriptional suppression and activation, as well as roles in nuclear organization, x-inactivation and splicing [13]. Interestingly, there is evidence from MeCP2 transgenic mice that mutations lead to neuropathological defects that include reduced brain size, motor dysfunction and seizures indicating an important role for these proteins in normal central nervous system development [12,13]. Studies utilizing MBD1 null mice have also indicated less severe neurological alterations including reduced neurogenesis in the hippocampus and deficits in spatial learning [13]. Transcriptional repression by MBD proteins, such as MBD2 and MeCP2, recruits histone deacetylase complexes (HDAC) and can be altered by HDAC inhibition [13,27,28]. Currently, it is proposed that MBD proteins, and in particular MeCP2, act both through recruiting enzymes that modify histone structure and by directly changing the structure of chromatin leading to a more condensed state [28].

Whereas Dnmt and MBD-modulated transcriptional repression may be long lasting and potentially permanent, in some cases modifications to the histone architecture appear to be a more dynamic epigenetic alteration of the transcriptome. Nucleosomes, which are composed of DNA wrapped around an octamer histone core, are the basic unit of chromatin [12]. Each histone has a N-terminal tail that projects outward, making it accessible to modifying enzymes that adjust the structure of the chromatin, depending upon the enzymatic process and the amino acid at which it occurs. These enzymatic alterations include methylation, acetylation, ubiquitination, phosphorylation and sumoylation, among others [8]. Whereas DNA methylation generally results in transcriptional suppression, histone methylation can be either transcriptionally permissive or repressive depending upon the type of histone, the location of the amino acid, and the number of modifications [10]. Histone acetylation is generally thought to be an activating mark that can lead to a relaxed chromatin state and increased transcription [10]. Histone deacetylation removes acetyl groups and can lead to a more condensed heterochromatic state suppressing transcription [10]. The histone code hypothesis postulates that modifications to histone tail residues may act in concert to create “on/off” switches that alter transcriptional tone through physical manipulation of the chromatin structure [29]. Specifically, when in the “on” state these changes in chromatin structure lead to enhanced transcription factor access to the DNA that is wound around the histone core. Conversely, in the “off” state, the histone architecture promotes more tightly wound DNA, leading to decreased transcription factor access and suppression of transcription. It has been hypothesized that histone modifications differ between the sexes, and in addition to DNA methylation, may serve an important function at certain stages of development or through influences on biological processes such as spermatogenesis [29]. There is evidence that males and females use different methods to inactivate X chromosomes in germ cells and that these strategies involve different histone modifications [30]. Depending on the developmental stage at which the alterations in chromatin structure occur, modifications may lead to neuroanatomical changes in the organism that last throughout its lifespan whereas other transcriptional changes may be temporary and affect behavior within specific circumstances.

Together, these epigenetic modifications potentially allow the prenatal or postnatal environment to influence the organism in the absence of genetic alterations. Epigenetic mechanisms can be transferred across generations or can have more immediate effects on the individual. Epigenetics can lead to sex differences in brain and body development [31] as well as sex-specific effects of environmental influences [32]. Sex differences in response to one such environmental influence—stress—will be explored in the course of this review.

### Epigenetic mechanisms and sexual differentiation of the developing brain

Epigenetic modifications lead to sex differences by shaping the neuroanatomy and steroid hormone receptor distribution of the developing brain. The neonatal brain is both masculinized and defeminized by a surge in gonadal hormones including testosterone [33,34]. This surge results from expression of the sex-determining region Y (SRY) gene on the Y chromosome, which drives testis development in the undifferentiated gonad. High levels of circulating testosterone released by the testis are then aromatized into estradiol within brain tissue and this in turn organizes the brain along a male to female continuum [35]. The female brain and body are prototypic and, in the absence of the surge in gonadal hormones, both brain and body will develop as female. Also present in females is a hormone-binding globulin, alpha fetoprotein, that protects the brain from masculinization by maternal estrogens [36].

One of the myriad effects of estradiol on the brain is alteration of the expression of estrogen receptor alpha (ERα) distribution in key brain regions linked to male sexual behavior such as the preoptic area (POA) [37]. ERα expression levels are decreased in males compared to females, and males show greater levels of DNA methylation at some CpG sites along the ERα promoter region [38]. Early postnatal (PND 2) estradiol exposure of female pups results in a masculine pattern of ERα distribution and promoter methylation status [38]. However, in disagreement with these previous findings, another lab exposing female pups to estradiol at the same time point reported increased methylation status of different CpG sites within the ERα promoter of the POA in females compared to males [39]. Therefore, it is likely a combination of activation and suppression at different sites on the promoter in males and females that leads to a more masculinized or feminized brain to influence sexual behavior.

Activation of ER during the prenatal period leads to the synthesis of prostaglandin E2 that in turn can permanently masculinize sexual behavior and the synaptic profile of the POA [40]. In addition DNA methylation, histone acetylation and deacetylation may also regulate sex differences in ERα receptor expression and brain masculinization. There are transient changes in the pattern of acetylation for histone 3 and 4 in the promoter regions of ERα and aromatase that differ in the male and female brain during prenatal development [40]. Exposure of male rats to an HDAC inhibitor decreased expression of male sexual behavior in adulthood. This maintenance of masculinization may occur through the actions of HDACs 2 and 4 as they bind with greater frequency to ERα and aromatase promoters in males than females [40].

Additional environmental factors such as maternal grooming also strongly influence ERα distribution and methylation of ERα promoter CpG islands [38]. Maternal rats normally engage in greater grooming of the male offspring’s anogenital region than female offspring [41]. Female pups that are exposed to simulated anogenital grooming show decreased ERα expression in the POA compared to non-manipulated females, and increased methylation of the ERα promoter [38]. It should be noted that in addition to acting epigenetically on the ERα in the POA, high attention maternal care can alter methylation of the glucocorticoid receptor (GR) in the hippocampus, and has been found to alter stress sensitivity in adult male offspring [42]. Therefore, this environmental factor may act epigenetically in multiple brain regions to alter sex specific sensitivity to stress, although this hypothesis has yet to be tested.

Even though alterations in the histone landscape may be transient, modifications occurring during critical developmental windows can permanently alter the brain. Males and females have different spatiotemporal patterns of histone methylation and acetylation in the cortex and hippocampus on embryonic day 18 and at birth [26]. Prenatal exposure to testosterone results in male-like patterns of acetylation, but not methylation, in the female cortex and hippocampus. Therefore, the combined effects of hormones and histone modifications are likely critically regulating transcription during this developmental window in brain regions associated with cognitive and emotional processing. Given that the sex differences in histone 3 lysine 9 (H3K9) acetylation continued to PND 6 but were not examined in adulthood, and that the study lacks behavioral endpoints, further research is needed to determine the role of this specific histone modification. However, other studies have demonstrated that the pattern of masculinization of the principal nucleus of the Bed Nucleus of the Stria Terminalis (BNSTp) is also dependent on a combination of testosterone exposure on PND1 and H3 acetylation [43]. Adult females have smaller BNSTp than males as a result of cell death starting on PND 6. Testosterone injection of females at birth can block this cell death and lead to a masculinized BNSTp in adulthood [44]. Application of an HDAC inhibitor to males, or injection of testosterone in females on PND 0, resulted in a feminized BNSTp in adulthood similar in size to control females. The BNST is necessary for the enhancing effects of acute stress on cognition in males [45] and masculinized females [44], but is not required for the detrimental effects of the same stressor in non masculinized females. Therefore, the epigenetic modifications associated with masculinization of this region may also contribute to developmental sex differences and the effects of stress upon cognition, although this has not yet been examined.

Both histone modifications and DNA methylation have been shown to alter sexually dimorphic brain structures during development. While these patterns of sexual dimorphism have been examined within the context of sexual behavior, there is a paucity of research on their functional role in the formation of sex differences in stress susceptibility. Future research should examine how epigenetic modifications that alter neuroanatomy affect vulnerability to stress in both sexes. 

### Epigenetic regulation of the behavioral response to gestational and transgenerational stress

Epigenetic modifications are also involved in an individual’s response to stress. This can be through developmental [46] and transgenerational effects [32], or through immediate effects on the organism [47] (Table 1). Variable stressor exposure of the mother during early gestation (PND 1–7) results in a dysmasculinzed behavioral stress response in male offspring [48]. As adults, these males show susceptibility to depression-associated behaviors, including increased time spent immobile in the tail suspension and forced swim tests, increased anhedonia, and increased wariness when investigating their environment. Further, these male offspring make cognitive behavioral choices more similar to those of females than control males [46,48]. Male offspring also show increased circulating levels of the stress hormone corticosterone (CORT) and decreased GR expression in areas of the hippocampus that undergo morphological alterations after exposure to stress [49,50]. Gestationally stressed males had increased corticosterone releasing factor (CRF) expression in the central nucleus of the amygdala that has the potential to contribute to dysregulation of the hormonal stress system [46] as well as changes in neurotransmitter release and circuitry with implications for other vulnerabilities such as addiction [51] and anxiety [52]. Examination of DNA methylation along the promoter regions for CRF and GR found that methylation was site specifically decreased in the CRF promoter, but increased in the GR promoter [46]. These data provide important correlational evidence that prenatal stress can epigenetically alter the stress system and its subsequent expression and function. These changes reflect alterations in behavior that affect offspring in a sex-specific manner. Some of the behavioral and transcriptional alterations manifest in second generation male mice (F2) bred from the prenatally stressed male offspring, indicating a hereditary epigenetic mechanism of transgenerational transmission [32]. In particular, transcription of HDACs was altered in male offspring bred from prenatally stressed males such that the transcriptional profiles of these offspring were more similar to females than control males.

**Table 1:** Epigenetic modifications associated with stress

**Stressor**	**Sex**	**Behavioral**	**Physiological**	**Epigenetic modifications**
		**effects**	**effects**	
Paternal Stress	Male and female	· Pro-depressant behavior in FST (both sexes) [53]	· Increased basal CORT (males) [53]	· Hypothesized but not examined.
		· Decreased sucrose preference (males) [53]	· Decreased VEGF (males) [53]	
		· Increased anxiety like behavior (both sexes) [53]		
		· Increased locomotor activity (males) [53]		
		· Increased social avoidance (males) [53]		
Gestational stress (PND 1-7)	Male and female	· Pro-depressant behavior in TST (males F1 [46] and F2 generation [32])	· Increased CORT response to stress (males-F1) [46]	· Decreased methylation of CRF promoter region [46]
		· Pro-depressant behavior in FST [46] (males)	· Increased CRF expression in central amygdala [46]	· Increased methylation in GR promoter region [46]
		· Altered navigation strategy for Barnes maze [48]	· Decreased GR in regions of hippocampus [46]	· Dysmasculinzed HDAC expression [32]
Early life stress (extended separation)	Male and female	· Pro-depressant behavior in FST [64]	· Increased HPA activity [64]	· Hypomethylation of Avp in hypothalamus. [64]
		· Decreased inhibitory avoidance [64]	· Decreased neurogenesis [67]	· Decreased MeCP2 binding in Avp enhancer [64]
		· Decreased spatial learning [65]		· Hypermethylation of Avp in hippocampus (males) [70]
				· Hypomethylation of NR4a1 in hippocampus (C57BL/6 males) [70]
Repeated Social defeat stress	Male	· Social avoidance [47]	· Changes in dendritic spine morphology in NAc [47]	· Dnmt 3a expression increased in NAc [47]
		· Decreased sucrose preference [54]	· Increased CRF expression in PVN of susceptible mice [22]	· Over expression of Dnmt 3a induced pro-depressant behaviors [47]
		· Increased anxiety [54]	· Decreased Bdnf in hippocampus [74]	· Blocking DNA methylation was antidepressant [47]
		· Alterations in metabolic function [54]		· Decreased methylation of the CRF promoter in PVN [22]
				· Increased repressive histone marks on BDNF promoters [74]
Sub chronic variable stress	Male and female	· Decreased sucrose preference (females) [56]	· Increased basal CORT (females) [56]	· Dnmt 3a, 3b and Dnmt1 expression increased in NAc [56]
		· Pro-depressant behavior in Splash test (females) [56]		· Altered in transcription for MBD proteins [56]
		· Decreased latency to eat in a novel environment (females) [56]		· Transcriptional and behavioral effects blocked by removal of Dnmt 3a (females) [56]
		· Pro-depressant behavior in FST (females) [56, 57]		

Another study examined transgenerational transmission of a stress- susceptible phenotype on the behavioral responses of the offspring of males exposed to repeated social defeat stress [53]. Repeated social defeat stress is an animal model of stress-related disorders that results in social avoidance behavior, anhedonia, metabolic dysfunction and increased anxiety associated behavior [54]. In this paradigm an experimental mouse is placed in the home cage of a larger mouse pre-selected for aggressive behavior. The animals are allowed to physically interact for 10 minutes before they are separated by a clear perforated divider that allows for continuous 24-hour sensory stimulus but prevents physical interaction. This process is repeated with a novel aggressor every day for 10 days [55]. Effects in experimental animals are long lasting and behavioral effects have been found at least 3 months following the social defeat stress [54]. Males that developed a depression-like phenotype, indexed by strong social avoidance behavior, were bred to females one month later. Using a sub-maximal social defeat protocol, designed to detect pro-susceptibility behavior, male offspring of defeated fathers showed enhanced social avoidance compared to the offspring of non-defeated controls.

Additionally, the authors compared the behavior of offspring bred prior to or post social defeat stress. They selectively found behavioral and hormonal changes in the male offspring bred post defeat. These included anxiety-associated behaviors such as decreased exploration of the open arms in an elevated plus maze, and increased depression-associated behaviors such as anhedonia and decreased latency to immobility in the forced swim test. Similarly, male offspring from defeated fathers had higher basal levels of the stress hormone corticosterone than offspring bred from fathers prior to defeat stress. Female offspring also exhibited some depression and anxiety-like phenotypes, including decreased latency to immobility in the forced swim test, and decreased time spent in the open arm of the elevated plus maze but in the absence of basal hormonal differences. To determine whether transmission of the stress effects occurred via epigenetic mechanisms in the sperm, the authors used *invitro* fertilization (IVF) techniques on defeated and control fathers. IVF was used to control for levels of maternal care, which can contribute to offspring stress responsiveness through epigenetic mechanisms [42], as the females mated with defeated males could potentially invest less maternal care in the offspring of these males than they would in the offspring of control males. While behavioral manifestations of the anxiety-associated phenotype did not transmit transgenerationally via sperm, the pro-depressant associated behavior in male and female offspring in forced swim test was replicated.

Maternal and paternal stress exposure is transmitted gestationally and transgenerationally through epigenetic mechanisms. Stressful experience by the parent may have a greater impact on stress responses in male offspring than female offspring, although more research is needed to directly test this hypothesis. The current data suggest that female vulnerability to stress changes across the lifespan. Females may be protected from gestational stress compared to males and yet may be more vulnerable to other forms of stress in adulthood [56-58]. However, the possibility also exists that current experimental strategies are unable to detect prenatal stress effects in females due to ceiling effects. The epigenetic mechanisms involved in the dynamics of female stress responses across the lifespan along with the potential for prenatal male vulnerability warrant further investigation. Additionally, the development of new behavioral techniques to examine sex differences in stress vulnerability is paramount so this issue can be adequately addressed.

### Epigenetic regulation of the behavioral response to early life stress

Early postnatal stress can lead to lifelong changes in numerous behavioral domains as well as stress reactivity, and these alterations are thought to involve epigenetic regulation. Maternal separation is one of the most common methods used to stress animals during early postnatal life [59]. Depending on the duration of the separation, both stress-susceptible and stress-resilient phenotypes can be observed. For example, daily maternal separation for 15 minutes can act to inoculate animals from stress leading to a resilient phenotype termed stress tolerance, which may promote active coping strategies [59,60]. Stress inoculated animals have reduced CRF expression in the paraventricular nucleus (PVN) of the hypothalamus along with reduced glutamatergic innervation of CRF hypothalamic neurons persisting into adulthood [60]. These effects coincide with a persistent increase in levels of the transcription factor neuron-restrictive silencing element (NRSF) that suppresses transcription of the CRF gene [60]. Additionally these animals have increased GR expression in the hippocampus and attenuation of the hormonal stress response [61-63]. Overall, these data indicate that short periods of early life maternal separation lead to a permanent decrease in excitability of the stress system through an enduring cascade of transcriptional events. Conversely, animals exposed to longer periods of early life stress (3 hours) display depression-associated behavior including greater time spent immobile in the forced swim test [64]. Additionally animals undergoing longer maternal separation display cognitive and memory deficits including altered inhibitory avoidance [64] and decreased spatial learning [65]. They have enhanced CORT production when exposed to a stressor [66] and have decreased cell proliferation in hippocampus leading to a smaller overall hippocampal volume [67]. Animals that are exposed to maternal separation in the early postnatal period followed by repeated social defeat in adulthood exhibit passive coping, displaying submissive postures and behavior [68]. Examination of epigenetic markers across the lifespan of animals exposed to early life stress found that the arginine vasopressin (*Avp*) enhancer in the PVN of the hypothalamus was consistently hypomethylated [64]. The hypomethylation was mediated by a loss of MeCP2 function, such that a specific CpG site in the enhancer region was not properly methylated. These animals exhibited consistent upregulation of *Avp* mRNA expression, along with increased basal levels of CORT and enhanced CORT release in response to stress. Like gestational stress, early life stress may have more robust behavioral and epigenetic effects on male rodents in adulthood [69]. A study that utilized a single 24-hour maternal separation paradigm found increased CORT levels in C57BL/6 males, but not females, following a swim stress in adulthood. This was mirrored by a greater methylation of the *Avp* gene in the hippocampus and decreased methylation of the *Nr4a1* gene, which encodes a nuclear receptor in the brain and has been implicated in depression and schizophrenia in humans [70]. These studies suggest that the same stressor may induce different epigenetic modifications in different brain regions, even when acting on the same genes. However, as different time periods of maternal separation were used, differences in methodology cannot be ruled out as a confounding causal factor. The second study also identified strain differences alongside sex differences in the ability of maternal separation to impact behavior and DNA methylation; therefore genetic background may also be a factor contributing to the different findings of these studies.

### Epigenetic regulation of the behavioral response to stress in adulthood

Although a number of chronic stressors have been used to induce depression-like behavior in adult rodents, few have been used to examine any associated epigenetic regulation. To date, the majority of studies examining stress in adulthood utilize repeated social defeat stress due to its face, predictive and construct validity. Repeated social defeat stress is not amenable to females, as they are less territorial than males and will not attack an intruding novel animal unless under special circumstances such as when nursing young [71] or through use of an aggressive territorial species known as California mice (Peromyscus californicus) [72]. Recent work has indicated a functional role for epigenetic modifications in susceptibility to stress. Repeated social defeat stress increases Dnmt 3a expression in the nucleus accumbens (NAc) [47], a brain region associated with emotional and reward processing [73]. Viral-mediated overexpression of Dnmt 3a in the NAc of adult male mice increased social avoidance behavior following a subthreshold microdefeat [47]. Overexpression of Dnmt 3a also decreased latency to immobility in the forced swim test [47]. Together these experiments indicate that overexpression of Dnmt 3a is sufficient to induce depression-associated behavior. Chronic intra-NAc infusion of RG108, a DNA methylation inhibitor, via osmotic minipump reversed social avoidance behavior in mice that had previously shown susceptibility to social defeat stress. These effects were similar to those produced by 28 days of systemic treatment with fluoxetine [47], indicating that blocking DNA methylation was antidepressant and suggesting that DNA methylation is necessary for engagement in social avoidance behavior.

DNA methylation of CpG islands along the CRF promoter in the PVN of the hypothalamus also coincides with the behavioral effects of social defeat stress. Animals that were susceptible to social defeat stress displayed higher levels of CRF expression in the PVN than animals that were resilient [22]. Susceptible mice also expressed decreased methylation of the CRF promoter at 4 specific CpG sites compared to resilient mice. Administration of an antidepressant blocked the development of social avoidance, as well as methylation of the CRF promoter. Social avoidance behavior in mice was also blocked using intra-PVN infusions of short interfering RNA (siRNA) sequences targeted to CRF. Viral-mediated gene transfer of the siRNA did not affect social interactions in control mice, but did attenuate social avoidance in animals exposed to repeated social defeat stress.

In addition to DNA methylation, other epigenetic mechanisms contribute to the behavioral manifestations of the social defeat stress. Chronic social defeat stress leads to decreased expression of two brain derived neurotrophic factor (Bdnf) transcripts in the hippocampus that coincides with repressive histone activity along the Bdnf promoter [74]. The behavioral effects of social defeat stress can be blocked using chronic antidepressant treatment, which also results in hyperacetylation of the Bdnf promoter, possibly counteracting the stress-induced repression. This hyperacetylation is functionally relevant as the behavioral effects of the antidepressant imipramine can be blocked by hippocampal-specific overexpression of HDAC 5 [74].

Studies have begun to examine other forms of stress during adulthood. Recent work reveals that both social stress and chronic restraint stress alters total methylation of the GR promoter 1_7_ in the adrenal and pituitary glands of young adult rats [75]. Methylation was decreased in the adrenal glands and increased in the pituitary, although there was no effect of either stressor on methylation of the same promoter in the hippocampus, PVN or cortex [75]. These animals did display altered CORT responses to an acute episode of restraint stress following the chronic stressor, suggesting that epigenetic modifications outside of the central nervous system may have functional relevance. Epigenetic modifications may be acting in concert in multiple brain regions and in the periphery to alter stress-induced behavior in adulthood. More work is needed to understand the functional significance of epigenetic modifications in relationship to stress and depression-related behaviors in adulthood, especially as females have been excluded from this line of research.

### Epigenetics and sex differences in the stress response

There is converging evidence that male animals are behaviorally more susceptible to prenatal and early life stress than females [76], whereas females are more susceptible to stress after puberty [77]. Therefore, it is unfortunate that studies of female stress during adulthood are so grossly underrepresented. The Russo laboratory is currently using a subchronic variable stressor (SCVS) to parse biological differences in the stress response [56,57]. After stress exposure females exhibit increased anhedonia, increased immobility in the forced swim test, increased latency to eat in a novel environment and decreased grooming behavior. While SCVS increases expression of Dnmt 3a in the NAc of both male and female mice, it is increased to a much larger extent in females compared to males [47,56]. Furthermore, removal of Dnmt 3a in the NAc of conditional floxed Dnmt 3a adult female mice blocks the behavioral effects of SCVS [56]. In addition to stress-induced elevation in Dnmt 3a transcription in the NAc, regulation is also observed for Dnmt 1, 3b and several MDB proteins and Tet 1. Transcription for all of these genes in the NAc are reversed in stressed females by removal of Dnmt 3a [56]. Therefore, we hypothesize that Dnmt 3a may regulate susceptibility to stress in a sex-specific manner. In our current model, we are examining whether pro-resiliency genes are methylated to a greater extent in females than they are in males. As discussed earlier in this review, the addition of stress leads to a signaling cascade that includes upregulation of DNA methylating enzymes and recruitment of MBD proteins and histones, which ultimately creates a more transcriptionally repressive chromatin structure. We hypothesize that females are more susceptible to stress because shorter periods of stress are necessary to induce suppression of resiliency genes due to existing methylation largely mediated by Dnmt 3a. We are currently in the process of testing this hypothesis and identifying potential resiliency genes.

## Conclusion

Given that epigenetic mechanisms contribute to both sexual dimorphism of the brain and susceptibility to stress, it is likely that there are sex specific modifications that shape responsiveness to stressful experience. As the clinical occurrence of depression is much higher in women, it is absolutely essential for the role of epigenetic modifications in stress related disorders to be examined in both sexes. Findings of significant differences in epigenetic programming between the sexes are rapidly emerging from developmental and transgenerational research that includes female subjects. A greater understanding of the mechanisms by which epigenetic modifications contribute to depression-associated behavior will help to clarify the etiology of mood disorders such as depression and anxiety. Additionally, this work will inform the identification of novel therapeutic targets for drug development. Already, HDAC inhibitors such as Valproic acid exist for the treatment of bipolar disorder. Unfortunately, these treatments are non-specific, and it is important to continue epigenetic research to yield novel treatments with fewer off-target side effects. Given the sex differences already found in epigenetic regulation of behavior, it is vital that any drug trials stemming from epigenetic research programs consider sex at both preclinical and clinical stages.

## Abbreviations

5-hmC: 5-Hydroxymethylcytosine; 5mC: 5-methylcytosine; Avp: Arginine vasopressin; Bdnf: Brain derived neurotrophic factor; BNSTp: Principal nucleus of the Bed Nucleus of the Stria Terminalis; CORT: Corticosterone; CpG: Cytosine- Phosphate- Guanine; CRF: Corticotrophin-Releasing Factor; DNA: Deoxyribonucleic acid; Dnmt: DNA methyltransferases; ER∝: Estrogen Receptor Alpha; FST: Forced swim test; GR: Glucocorticoid receptor; H3K9: Histone 3 Lysine 9; HDAC: Histone deacetylase complex; IVF: In vitro fertilization; MBD proteins: Methyl-CpG binding domain proteins; MeCP2-Methyl: CpG binding protein 2; NAc: Nucleus Accumbens; NRSF: Neuron-restrictive silencing element; PND: Post Natal Day; POA: Pre Optic Area; PVN: Paraventricular Nucleus; SCVS: Sub Chronic Variable Stress; SiRNA: Short Interfering Ribonucleic Acid; TST: Tail suspension test.

## Competing interests

The author has no competing or financial interests to disclose.

## Author’s contribution

GEH wrote and edited the manuscript.
